# Heparan sulphate and neural development: dissecting the roles of astrocyte-expressed heparan sulphate

**DOI:** 10.1042/BST20253088

**Published:** 2025-12-24

**Authors:** Martina Gyimesi, Rachel K. Okolicsanyi, Larissa M. Haupt

**Affiliations:** 1 Stem Cell and Neurogenesis Group, Genomics Research Centre, Centre for Genomics and Personalised Health, School of Biomedical Sciences, Queensland University of Technology (QUT), 60 Musk Ave., Kelvin Grove, Queensland (QLD), 4059, Australia; 2 Max Planck Queensland Centre for the Materials Sciences of Extracellular Matrices, Queensland University of Technology (QUT), 60 Musk Ave., Kelvin Grove, Queensland, 4059, Australia; 3 ARC Training Centre for Cell and Tissue Engineering Technologies, Queensland University of Technology (QUT), Australia

**Keywords:** astrocytes, brain development, neurodegeneration, neurogenesis, proteoglycans

## Abstract

Astrocytes are key regulators of neurogenesis, synaptogenesis, synaptic transmission and the clearance of pathological factors within the brain, while maintaining homeostasis throughout life. They also aid in the establishment and maintenance of a neurogenic niche enriched with precisely balanced growth factors, morphogens and extracellular matrix proteoglycans (PGs) to support neuronal development and function. Membrane-bound heparan sulphate (HS) PGs consist of core proteins decorated with HS glycosaminoglycan side chains, whose highly variable sulphation patterns regulate cellular signalling pathways such as Wnt and fibroblast growth factor. However, the specific contributions of astrocyte-derived and/or neuronal HSPGs within this microenvironment remain unclear. This mini-review examined our current understanding of the regulatory role of astrocyte-expressed HSPGs and their associated HS side chain structural variability. In particular, their influence on prenatal brain development, ageing and the changes occurring that contribute to neurodegeneration. We focused on the emerging concept that HS aggregation and impaired neurogenesis may serve as important preclinical contributors to Alzheimer’s disease pathology. Alterations in astrocyteexpressed HS and their HSPG landscape are discussed as potential precursors to pathological HS aggregation and reactivity, shifting the focus of disease initiation to the potential compromise of the supportive astrocytic environment. We suggest that neuronal dysfunction cannot be solely attributed to neurodegeneration but must also be considered in the context of a deteriorating support system, where cells that once nurtured neurogenesis and synaptic integrity become dysfunctional contributors to disease pathology.

## Introduction

Understanding the human brain and its intricacies remains one of the greatest challenges in modern science and medicine. From the formation of the neural tube in the embryo to the final stages of life, numerous signalling pathways regulate essential brain processes, including the sequential differentiation of stem cells into various neural subtypes [1–3], the formation and recollection of memories [4,5] and the organised clearance of pathological factors [6,7]. These processes are orchestrated through signalling mechanisms via direct cell-to-cell interactions between receptor-expressing and ligand-expressing cells, as it occurs in the Notch signalling pathway [8], or in response to secreted growth factors and morphogens within the extracellular environment, as exemplified by the canonical Wnt signalling pathway [9]. Astrocytes, a major glial cell type, are central to supporting these processes through maintenance of neurotransmitter balance, along with modulation of signalling molecules for neural repair or ligand expression for direct activation of neural differentiation of neural progenitor cells (NPCs) [10–12].

The specialised extracellular environment supporting the differentiation of neural subtypes is termed the neurogenic niche and comprises neurones, glial cells and vasculature. Unlike most adult brain regions, which do not support constitutive neurogenesis, these niches are exceptional in their ability to maintain neural stem cells. In humans, these highly specialised microenvironments are located within the subgranular zone (SGZ) of the dentate gyrus and the subventricular zone (SVZ) [13]. The niche is supported by a dynamic extracellular matrix (ECM) combining fibrous proteins such as collagens and elastins, growth factors, cytokines and a diverse array of proteoglycans (PGs) and glycosaminoglycans (GAGs) [14], some of which can form perineuronal nets to support mature neurones. The specific type of GAG side chains (chondroitin, dermatan, HS or keratan sulphate) determines the regulatory roles and specific interactions of their associated PGs. Here, we focused on HS, specifically in regard to membrane-bound HSPGs, key mediators of neural lineage specification [15,16]. Of particular interest are the membrane-bound syndecans (SDCs) and glycosylphosphatidylinositol-anchored glypicans (GPCs), expressed and secreted by astrocytes and their effects on the neural niche and during neurodegeneration.

**Figure 1:**
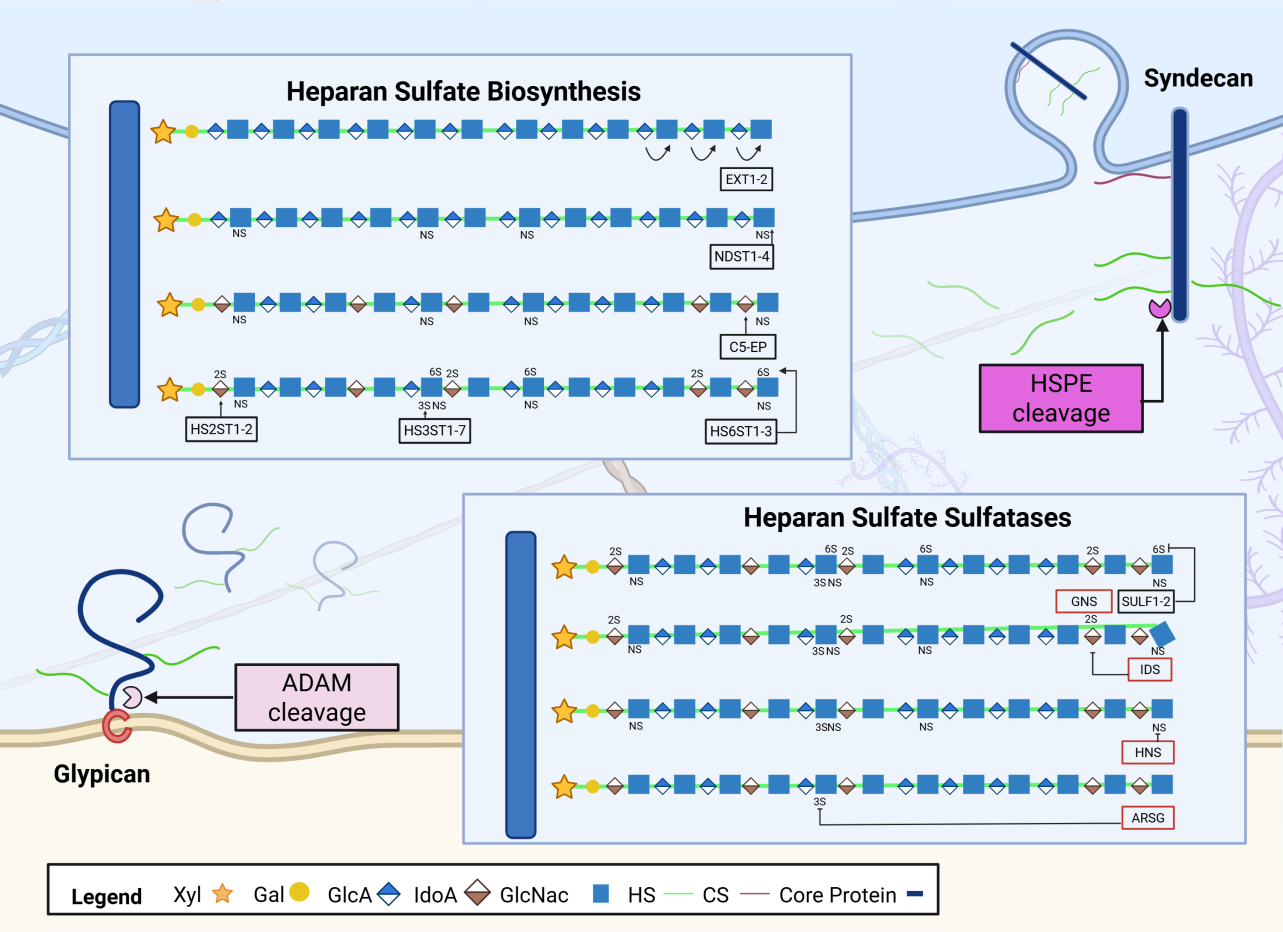
Heparan sulphate biosynthesis and degradation. This figure illustrates the key biosynthetic steps of heparan sulphate (HS) assembly on heparan sulphate proteoglycans (HSPGs), together with selected modifying and degradation enzymes, with particular focus on sulphatases. This figure focuses on transmembrane syndecans (SDCs) and GPI-anchored glypicans (GPCs), highlighting the role of heparanase (HPSE) in HS cleavage and A D isintegrin A nd M etalloprotease (ADAM) proteases in HSPG ectodomain shedding. The HS biosynthesis in this illustration begins with exostosin 1/2 (EXT1/2) adding N-acetylglucosamine (GlcNAc) residues to the growing glycosaminoglycan (GAG) chain. N-deacetylase/N-sulphotransferases (NDSTs) catalyse N-sulphation (NS), whereas C5-epimerase (**C5-EP**) mediates the epimerisation of glucuronic acid (GlcA) to iduronic acid (IdoA) within HS domains. This process enables further sulphation modifications, including 2-O-sulphation by heparan sulphate 2-O-sulphotransferase (HS2STs), 3-O-sulphation by heparan sulphate 3-O-sulphotransferases (HS3STs), and 6-O-sulphation by heparan sulphate 6-O-sulphotransferases (HS6STs). These sulphation patterns can then be selectively removed by extracellular sulphatases (which remove 6-O-sulphate groups; SULFs) and lysosomal sulphatases (N-acetylglucosamine-6-sulphatase – GNS) that are marked by red. Further, iduronate-2-sulphatase (IDS) removes 2-O-sulphation, heparan N-sulphatase (HNS) removes N-sulphation, and arylsulphatase G (ARSG) removes 3-O-sulphation. NS, N-sulphation; IdoA, iduronic acid; CS, chondroitin sulphate; HNS, heparan N-sulphatase; C5-EP, C5-epimerase; ARSG, arylsulphatase G; IDS, iduronate-2-sulphatase; HPSE, heparanase; GNS, N-acetylglucosamine-6-sulphatase; GPI, glycosylphosphatidylinositol; HS2STs, heparan sulphate 2-O-sulphotransferase.

HSPGs are ubiquitous glycoproteins mediating cellular signalling pathways through interactions with fibroblast growth factor 2 (FGF2), Wnt ligands and bone morphogenic proteins (BMPs) [17–20]. These interactions are determined by their highly variable and dynamically responsive HS sulphation profile created by a complex and temporal biosynthesis process within the Golgi apparatus [21]. The initiation of HS chain synthesis involves the assembly of a linkage tetrasaccharide on the core polypeptide, catalysed by four enzymes, including O-xylosyltransferases (XYLT1-2), galactosyltransferases (GalT-1–2), and glucuronyltransferase (GlcAT-1) [21]. This is followed by the addition of a single N-acetylglucosamine (GlcNAc) unit to the non-reducing end of the growing chain by one or more α-GlcNAc transferases [Exostosin-like (EXTL)]. The polymerisation of the HS chain occurs through the addition of alternating glucuronic acid (GlcA) and GlcNAc residues, catalysed by the exostosin glycosyltransferase family proteins (EXT1-2) [21]. During the polymerisation process, HS chains undergo multiple modifications, including N-deacetylation and sulphation [N-deacetylase/N-sulphotransferase 1-4 (NDST1-4)], C5 epimerisation [Glucuronic Acid Epimerase (GLCE)] and variable O-sulphation at specific sites [heparan sulphate 2-O-sulphotransferase 1 (HS2ST1); heparan sulphate 6-O-sulphotransferases 1-3 (HS6ST1-3); heparan sulphate 3-O-sulphotransferases 1-7 (HS3ST1-7)] [21] (Figure 1).

In this mini-review, we explored prenatal brain development and adult neurogenesis through the lens of glia and HSPGs with an aim to delineate their associated glial-specific dysfunction resulting in the neurodegenerative disorder Alzheimer’s disease (AD). While reactive astrocytes are widely recognised in AD, they are currently regarded as primarily responsive to amyloid and tau pathologies. We suggest changes to astrocytes and their role in supporting and mediating neurogenesis occur prior to their transition into a reactive state. This provides a potentially new perspective on AD and neurodegenerative pathology, specifically, through a deeper examination of the often-underexplored contribution of astrocytes to neural development, neurogenesis and neuroprotection.

### Early brain development

Human prenatal brain development begins approximately three weeks into gestation with the differentiation of the ectoderm into the neural plate [22]. This structure folds to form the neural tube [23], which later develops into the brain – a process that continues with active synaptic remodelling until around 25 years of age [24]. During embryogenesis, radial glial cells act as NPCs capable of generating both neuronal and glial lineages.

Glial cell development primarily occurs during late gestation and postnatal stages in mammals, with early and mid-gestation predominantly marked by neuronal differentiation [25,26]. To understand the factors necessary for gliogenesis, this developmental trajectory can be mirrored in studies of human foetal brains. As an example, an online human developmental biology expression resource revealed an intriguing interplay between HS sulphation enzymes from 40 to 140 days post-conception (dpc) [27]. While late gestation, typically defined as around 168 dpc in humans, is not represented in the study, the data provides valuable insight into the contribution of HS sulphation enzymes during neurogenesis and the priming of gliogenesis at the early stages of development [28]. The study demonstrated low expression levels of NDST2 and HS3ST3 throughout the 100-day timeline (40–140 dpc), with NDST4 and HS3ST2 expressed exclusively during the very early pc stages, suggesting specific activity for these biosynthetic enzymes during neuronal development [27]. Interestingly, HS3ST1 expression was found to increase later, around 100 dpc, potentially indicating a specific role for 3-O sulphation in priming for gliogenesis [27]. Although increased expression of HS3ST1 does not necessarily correspond to higher overall levels of 3-O sulphation [29], early studies demonstrated that spatially and temporally distinct HS3ST isoforms generate unique 3*-*O-sulphated domains, which may be required for the specific signalling interactions needed for gliogenesis [30]. For example, early studies in *Drosophila* showed that HS 3-O sulphation activates the Notch signalling pathway, critical for glial cell differentiation [31,32].

Interestingly, lysates from primary rat astrocytes from gestational day 21 (late gestational period in rats [33]) showed high levels of mostly unsulphated HS-GAGs [34]. The significantly higher levels of HS-GAGs observed in cortex lysates further suggest astrocytes to be the primary source of HS in the developing brain [34]. Notably, these HS molecules may lack sulphation due to the significantly lower observed level of *NDST3* and *NDST4* in prenatal astrocytes [34]. While NDST1 and NDST2 are widely expressed throughout the body, early studies have shown NDST3 and NDST4 to exhibit brain-specific expression, with NDST3 possessing high N-deacetylase activity and NDST4 demonstrating strong N-sulphotransferase activity [35]. Given the temporally regulated nature of HS biosynthesis, the absence of specific NDST isoforms is likely to result in reduced N-sulphation of the HS side chains, critical for facilitating subsequent 6-O and 3-O sulphation events during later stages of modification [36,37]. However, this lack of sulphation may also serve a functional purpose by allowing astrocytes to dynamically remodel sulphation patterns in response to environmental cues. Such flexibility could be crucial for regulating neurogenesis, synaptogenesis and response to neuroinflammation.

### Developmental disorders, HS and astrocytes

Developmental disorders of the brain often arise from disruptions to critical cellular processes, including impaired signalling pathways, genetic mutations or environmental insults [25]. While many neurodevelopmental disorders have been attributed to aberrant neurogenesis, synaptogenesis or neural circuit formation, it is increasingly recognised that these processes are mediated, in part, by astrocytes [25,38]. Disorders such as autism spectrum disorder (ASD), attention deficit hyperactivity disorder (ADHD) and epilepsy are among those that have been linked to astrocyte dysfunction [39]. For example, reduced levels of HS in the SVZ have been observed in postmortem brain tissue from young to mature individuals with ASD in comparison with age-matched, typically developing controls [40]. Notably, this reduction was concurrent with a decreased number of glial fibrillary acidic protein (GFAP)/S100 calcium-binding protein B (S100B)-positive astrocytes, as shown by a separate study in post-mortem ASD individuals [41].

Conversely, increased numbers of astrocytes have been documented in studies exploring epilepsy-prone brain regions, including the hippocampus and cortex [42], and more recently have subsequently been observed in the temporal lobe of epilepsy patients [43,44]. While no recent studies have correlated increased HS in epilepsy, early findings examining mucopolysaccharidoses in patients identified elevated brain HS to be associated with a higher incidence of seizures [45].

Interestingly, the number of astrocytes also influences the secretion of astrocyte-derived factors such as GPC4, which promotes excitatory synapse formation through α-amino-3-hydroxy-5-methyl-4-isoxazolepropionic Acid (AMPA) glutamate receptor activation [46–48]. AMPA receptor hyperactivity has been implicated in epilepsy, with the use of inhibitors showing therapeutic potential [49]. As such, it is plausible that increased astrocyte numbers could elevate GPC4 secretions to modulate AMPA receptor activity and contribute to hyperexcitability, although direct evidence of this in epilepsy remains limited. In contrast, the absence of GPC4 has been identified to reveal age-dependent behavioural alterations similar to ASD in GPC4 knockout (KO) mice [50]. Juvenile GPC4 KO mice displayed hyperactivity in open-field tests, while adult GPC4 KO mice exhibited deficits in social novelty behaviours, with non-social behaviours such as working memory and anxiety unaffected. These behavioural changes may be explained by disrupted synaptic glutamate receptor subunit 1 levels, further contributing to ASD-associated phenotypes [50].

It is important to note that distinguishing astrocyte-secreted GPC4 from contributions of other cell types is challenging, given the widespread abundance of HSPGs throughout the brain and body. When referring to astrocytic GPC4, studies have typically relied upon co-immunostaining with astrocyte markers or purified cultures [48], as well as single-cell sequencing or cell-type-specific tagging approaches [47], with each of these methods limited by resolution, specificity or capture of the *in vivo* complexity. The direct attribution of secreted HSPGs to astrocytes when compared with neurones *in vivo* remains extremely difficult. Once released into the extracellular space, HSPGs mix and interact within the shared ECM, making their cellular origin hard to resolve even with single-cell transcriptomics or tagging strategies. As a result, most evidence to date has relied on inference from *in vitro* cultures or cell-type specific knockout models (e.g. astrocytic *GPC4* deletion).

### HS in adult gliogenesis

**Figure 2:**
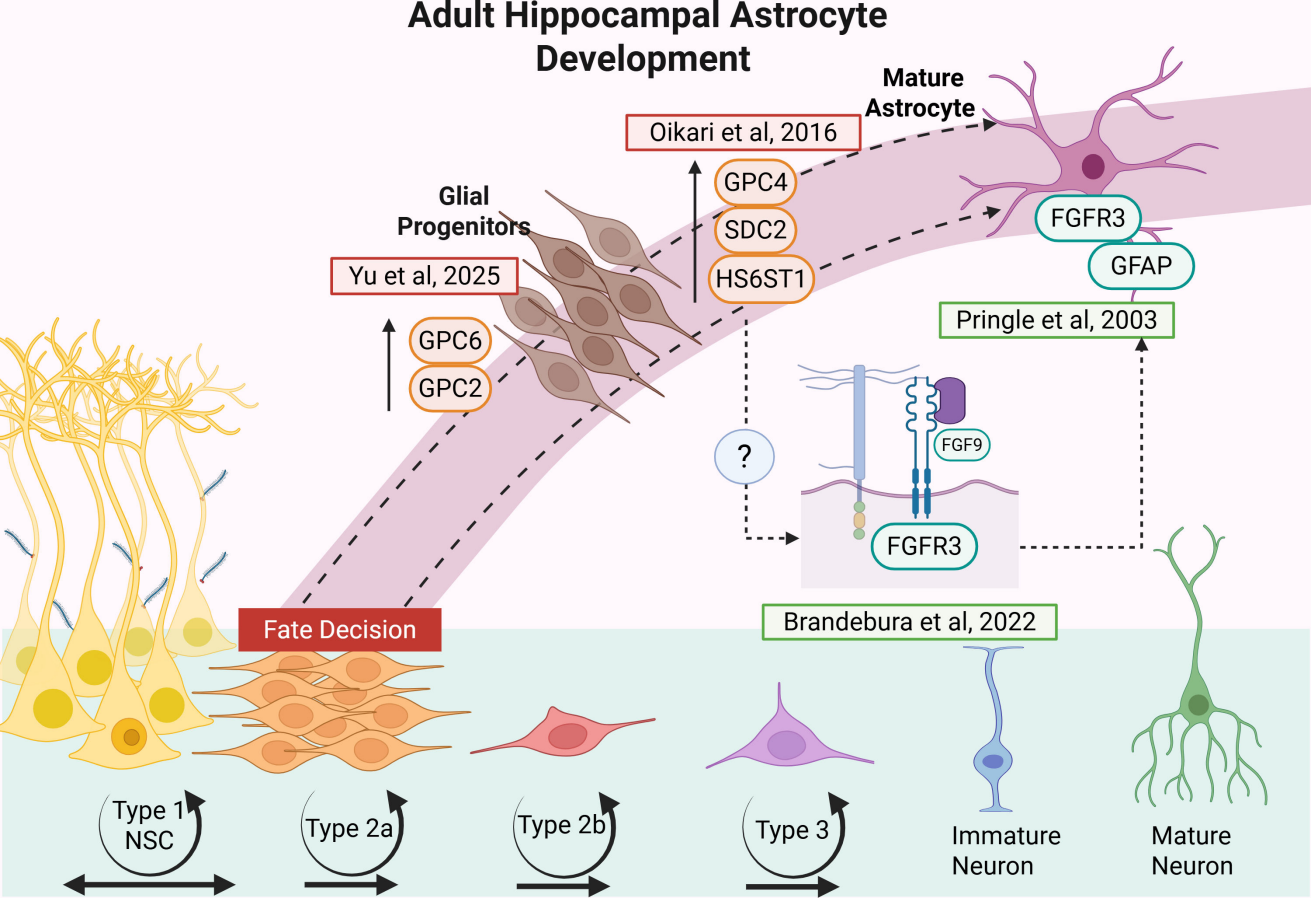
Neuro- and gliogenesis in the subgranular zone of the hippocampus. This schematic illustrates the development of mature neurones and astrocytes from type 1 neural stem cells (NSCs) in the subgranular zone, with emphasis on heparan sulphate proteoglycan (HSPG) expression patterns. Type 1 NSCs exhibit high self-renewal capacity (circular arrows) and can generate either quiescent NSCs (not shown) or type 2a neural progenitors. Type 2a cells undergo a critical fate decision: they may commit to the neuronal lineage, progressing through type 2b and type 3 progenitors to become mature neurones, or alternatively, adopt a glial fate. Yu et al. [55] reported increased expression of glypican 6 (GPC6) and GPC2 in induced astrocytes derived from neural progenitor cells, although these *in vitro* astrocytes are unlikely to fully match the properties of primary astrocytes *in vivo*. Similarly, Oikari et al. [56] observed up-regulation of GPC4 and SDC2 in long-term astrocyte-differentiated hNSC-H9 cells, which may represent a more mature state, though still not validated *in vivo*. Both studies were conducted in human cell systems. In contrast, murine studies have shown that fibroblast growth factor receptor 3 (FGFR3) and FGF9 co-operate to promote astrocyte maturation and GFAP expression, suggesting a possible regulatory role of HSPGs through 6-O sulphation [57,58]. While this remains speculative, it underscores the need for studies using longer or more mature primary astrocyte cultures to better define HSPG expression patterns in the late stages of gliogenesis *in vivo.* NSCs, neural stem cells.

Astrocytes continue to be generated postnatally through gliogenesis, which occurs exclusively in the SGZ of the hippocampus and the SVZ of the lateral ventricles [51]. Gliogenesis begins with the proliferation of human neural stem cells (hNSCs), regulated primarily by Notch and Wnt signalling pathways, followed by asymmetric division into type 2 cells (types 2A and 2B) [1,52,53]. These progenitors express nestin (NES) and SRY-Box Transcription Factor 2 (SOX2) but lack GFAP expression [54]. Early murine studies have shown that at the type 2A stage, a critical cell fate decision determines whether NPCs differentiate into the glial or neuronal lineages [54](Figure 2).

A more recent study demonstrated that BMP4, transforming growth factor beta 2 (TGFβ2), neuroligin 1, thymic stromal lymphopoietin and Dickkopf-1 (DKK1) synergistically direct this switch from neurogenesis to gliogenesis through signal transducer and activator of transcription 3 (STAT3) and small mothers against decapentaplegic (SMAD) activation [59]. These transcription factors bind directly to *GFAP* promoters, as shown in embryonic mice-derived neural epithelial cells [60]. While the study did not explore HSPGs, it is well-established that HSPGs are key receptors for the BMP, TGFβ and Wnt pathways [61]. Key examples of this include GPC1 and GPC3 inhibition of BMP4 in human primary suture mesenchymal cells [62] and SDC2 regulation of TGFβ2 signalling via SMAD pathways in human fibrosarcoma cells [63]. *GPC4* loss of function mutants in zebrafish have been identified to increase canonical Wnt signalling and to be rescued by the inhibitory effects of DKK1, suggesting functional similarity between DKK1 and GPC4 [64]. Although these early studies were performed in non-neural cell types, they demonstrate direct HSPG involvement in BMP, TGFβ and Wnt signalling pathways that may be relevant to gliogenesis and specifically the neurogenesis-to-gliogenesis switch. However, extrapolating findings from non-neural tissues to neural contexts and vice versa has been historically demonstrated to be problematic [65]. More recently, *in vitro* models of human neurogenesis have begun to be explored to investigate how HSPGs and their HS side-chain structures influence lineage specification. Since directly tracking the neurogenesis-to-gliogenesis switch *in vivo* remains technically and ethically challenging, current studies and evidence rely largely on 2D and 3D *in vitro* approaches.

As such, early studies examining *in vitro* differentiation of hNSCs in astrocytic lineage inductive culture conditions resulted in up-regulation of *NDST3, HS6ST1*, *SDC2* and *GPC4* [56]. This is in contrast with the levels observed in basal embryonic hNSC-H9 cells and neuronal derivatives, where GPC1 and GPC3 increased only in hNSC neuronal inductive lineage differentiation culture conditions [56]. While HS6ST1-mediated 6-O sulphation is critical for Wnt ligand binding, it is also relevant to FGF1 and FGF9 signalling as demonstrated in rat astrocytes [66]. Notably, in mice, neuron-derived FGF9 interaction with FGFR3 (often co-expressed with GFAP *in vitro* and *in vivo* in the mouse central nervous system [57]) was found to be necessary for promotion of astrocyte maturation through regulation of GFAP expression [58]. In murine osteogenic MC3T3-E1 cells, FGFR3 has also been found to be associated with GPC3 expression and terminal differentiation [67]. While GPC3 expression was not elevated in astrocyte-differentiated NSCs, it may mediate lineage commitment at earlier stages, particularly in type 2A progenitor cells. A more recent study identified GPC2 and GPC6 as contributors to astrocyte lineage commitment in early stage astrocyte-inductive immortalised human induced NPCs (ReNcell CXs) [55] and confirmed the association of HS6ST with astrocyte differentiation in the same system. Theoretically, increased HS6STs would primarily increase the prevalence of 6-O groups; however, mast cell HS6ST1 KO studies identified no difference in the amount of 6-O sulphation on heparin/HS, with increased N-S and 2-O sulphation patterns observed [68]. These enzymes and resulting sulphation patterns likely facilitate astrocyte function through modulating interactions with key signalling molecules, particularly Wnts (which have a high affinity for 6-O sulphation) and FGFs (which preferentially bind to 2-O sulphation) [69,70]. These interactions are essential for maintaining the balance between proliferation and differentiation of NSCs with the differential response to Wnt and FGF signalling, dependent on the combined presence of 6- and 2-O sulphation sites on the GAG chains [71].

It is worth noting that although several studies have examined astrocyte induction *in vitro* using human cell models, there are limited current studies specifically investigating HSPG expression or co-expression in human brain tissue or mature primary astrocyte cell cultures. This remains a clear gap in our understanding, particularly as much of what we know about neuro/gliogenesis has historically been derived from murine and other animal models. While astrocyte-induced stem cell cultures offer a valuable platform with which to monitor molecular and cellular changes, the differentiated populations generated are unlikely to fully recapitulate the phenotype and function of mature astrocytes as they occur *in vivo*. While changes in gene and protein expression, as well as potential molecular interactions, are valuable observations in mouse and stem cell models, it remains essential to validate these findings in relevant tissue and/or primary astrocyte cell cultures. This is particularly important, as HSPGs within the local microenvironment may represent key targets in the regulation of the shift from neurogenesis to gliogenesis*.*

### Alzheimer’s disease and reactive astrocytes

Our understanding of AD has long been informed by early studies characterising dysregulated amyloid precursor protein (APP) processing, leading to the formation of amyloid beta (Aβ) plaques and tau tangles, resulting in neuronal atrophy [72]. Pathological stimuli act as triggers for astrocyte activation, leading to morphological, structural and functional remodelling [73]. This process ultimately results in astrocyte overproliferation, increased cytokine release and a shift from homeostatic function to an emergency immune response [74]. The now reactive astrocytes exhibit a dual role in AD: (i) promotion of neuroprotection via clearance of pathological factors and release of neurotrophic factors; and (ii) contribution to neurodegeneration through the release of pro-inflammatory cytokines and reactive oxygen species [75]. While the contribution of astrocytes to AD pathology is well-established and will be briefly addressed, changes in astrocytes, their associated HS landscape and their contribution to the onset of AD-related pathological factors warrant further scrutiny.

Given the high abundance and functional versatility of HS and HSPGs within the brain, it is unsurprising that these ubiquitous proteins interact with both Aβ plaques and tau proteins, contributing to AD pathogenesis [76]. HS sulphation patterns are key endogenous regulators of APP processing, influencing Aβ and tau internalisation through SDC4 and 3-O sulphation motifs, respectively [77–81]. Interestingly, SDC4 has also been implicated in astrocyte-neurone interactions through inhibition of neurite extension and induction of neurite retraction and found to be overexpressed in AD transcriptomic studies [82–84]. Whether this overexpression is a response to the increased need for extracellular Aβ endocytosis or an early event resulting in dysfunctional astrocyte-neurone interactions is not known.

However, reactive astrocytes have been observed in injured mouse brains to up-regulate SDC1 and SDC3 expression, along with APP, in response to pathological microenvironmental changes [85,86]. Studies have indicated SDCs preferentially bind to apolipoprotein E (ApoE), a glycosylated protein involved in Aβ aggregation and clearance under normal conditions [87,88]. While the precise interactions in early AD development remain unclear, the high-risk ApoE4 isoform has been identified to have a threefold higher affinity for HS and association with cognitive decline [89,90]. Notably, complete depletion of astrocytic ApoE through astrocyte knockout studies rescued Aβ aggregation [91,92]. Although ApoE binding to SDCs may reduce Aβ clearance, this mechanism has not been directly demonstrated and remains speculative.

## Utilising HS in a biologically relevant *in vitro* model of reactive astrocytes

Modelling preclinical AD remains a challenge due to the complexity of its early pathophysiological drivers. A critical gap in our understanding lies in how astrocytes and their developmental and microenvironmental maintenance trajectories contribute to an environment permissive of AD pathogenesis. Rather than viewing AD solely as a failure of neurogenesis, we propose that an imbalance between neurogenesis and gliogenesis disrupts the tightly regulated and highly specific ratio of HS and HSPG at critical neural lineage and maturation stages. This dysregulation alters signalling pathway activation, shifting developmental trajectories to promote an environment that fosters neurodegeneration. To investigate this, we propose leveraging HS and HSPGs as the foundational axis from which to develop biologically relevant *in vitro* models.

A clearer understanding of HS-dependent regulation of glial fate may be gained through the examination of HS biosynthesis enzymes and specific HS motifs in single-cell type cultures to determine their contribution to NSC priming toward astrocyte-specific lineage differentiation. This could be achieved through advanced mass spectrometry approaches, such as spatial glycoproteomics and disaccharide compositional analysis, to define HS chain structures and sulphation patterns at key stages [93,94]. Integration of phenotypic data with single-cell RNA sequencing or spatial transcriptomics will allow mapping of how these HS modifications drive neural fate in normal and pathological conditions.

Astrocyte reactivity, a hallmark of AD, has been linked to excessive activation of epidermal growth factor (EGF) receptors, driven in part by heparin-binding EGF-like growth factor (HB-EGF) [95]. *In vitro* systems incorporating exogenous heparin may provide critical insights into the mechanism of HS in mitigating HB-EGF-induced astrocyte reactivity, potentially revealing dose-dependent effects to mirror early pathogenic AD processes. Furthermore, examination of the functional contributions of short, highly sulphated HS fragments secreted by glial progenitors may further clarify their role in gliogenesis and whether altered HSPG expression may skew lineage commitment.

In addition, a more detailed exploration of conditioned media from a range of 2D and 3D cultures (monocultures, patient-derived or immortalised AD glial and neuronal cells) via using targeted liquid chromatography–tandem mass spectrometry may distinguish HS fragments derived from specific cell types. Modulating HS biosynthesis through knockdowns or enzymatic inhibition (e.g. chlorate treatment) will also enable more detailed delineation of sulphation-dependent processes, while the use of lithium chloride may uncover HS and HSPG functions independent of canonical Wnt signalling, possibly through interactions with TGFβ and Notch pathways [96].

Given the temporal regulation of HS and HSPG biosynthesis, cellular responses are likely dose-dependent, providing an opportunity to map enzyme utilisation and oscillatory expression patterns during different stages of gliogenesis. A key limitation to this is that HS biosynthetic enzyme expression does not necessarily correlate with HS side chain structure. Therefore, changes in gene or protein expression should not be assumed to reflect alterations in HS structure.

While gene knockdown and siRNA-based approaches are invaluable in establishing mechanistic insights, they fail to replicate the complexity of *in vivo* pathology. A more physiologically relevant approach would involve leveraging the inherent differences in HSPG expression across neural lineages and maturation stages. Rather than relying solely on genetic perturbations, co-culturing distinct lineage-specific populations may offer deeper insights into HSPG-mediated regulation, allowing for a more refined exploration of cell–cell interactions and endogenous regulatory mechanisms that drive gliogenesis and astrocyte reactivity.

The continued development of biologically relevant 3D culture systems that build on established 2D data in human cell models cannot be overstated, with transcriptomic and proteomic profiles being highly context-dependent. 3D cultures provide a spatially relevant framework to assess cellular interactions, localisation and the transfer of pathological factors in co-culture systems, allowing for a more accurate assessment of whether excessive gliogenesis disrupts neuronal function and promotes astrocyte reactivity.

A comprehensive evaluation of HS sulphation dynamics, secretion patterns and downstream effects on gliogenesis may uncover novel regulatory mechanisms contributing to AD pathology. By reframing HSPGs not simply as passive extracellular components but as dynamic mediators of neurodevelopment, we may reveal new mechanistic insights with therapeutic implications not only for AD but also for a broader spectrum of neurodegenerative and neurodevelopmental disorders.

**Perspectives**

- **Importance of the field**: Astrocytes and their heparan sulphate proteoglycans (HSPGs) are critical regulators of neurogenesis and gliogenesis, with implications for both brain development and neurodegenerative disease.
- **Current thinking**: Dysregulation of HS/HSPG expression in astrocytes may precede reactive states and contribute to Alzheimer’s disease pathology by altering neurogenic niches and cell signalling.
- **Future directions**: Studies using primary human astrocytes, advanced *in vitro* models and single-cell glycoproteomics are needed to define HS structural dynamics and their functional roles in neural lineage specification and disease progression.

## Abbreviations

AD: Alzheimer’s disease
AMPA: α-amino-3-hydroxy-5-methyl-4-isoxazolepropionic acid
APP: amyloid precursor protein
ApoE: apolipoprotein E
Aβ: amyloid beta
BMPs: bone morphogenic proteins
DKK1: Dickkopf-1
ECM: extracellular matrix
EGF: epidermal growth factor
FGF: fibroblast growth factor
GAGs: glycosaminoglycans
GFAP: glial fibrillary acidic protein
GPCs: Glycosylphosphatidylinositol-anchored glypicans
GlcNAc: N-acetylglucosamine
HB-EGF: heparin-binding epidermal growth factor-like growth factor
HS: heparan sulphate
HSPGs: heparan sulphate proteoglycans
HS3STs: heparan sulphate 3-O-sulphotransferases
HS6STs: heparan sulphate 6-O-sulphotransferases
KO: knockout
NDSTs: N-deacetylase/N-sulphotransferases
NPCs: neural progenitor cells
PGs: proteoglycans
S100B: S100 calcium-binding protein B
SDCs: syndecans
SGZ: subgranular zone
SMAD: small mothers against decapentaplegic
SOX2: SRY-Box Transcription Factor 2
STAT3: signal transducer and activator of transcription 3
SVZ: subventricular zone
TGFβ2: transforming growth factor beta 2
dpc: days post-conception
hNSCs: human neural stem cells
